# Effectiveness of high-flow nasal cannulae compared with noninvasive positive-pressure ventilation in preventing reintubation in patients receiving prolonged mechanical ventilation

**DOI:** 10.1038/s41598-023-31444-8

**Published:** 2023-03-22

**Authors:** Chi-Wei Tseng, Ke-Yun Chao, Hsiu-Li Wu, Chen-Chun Lin, Han-Shui Hsu

**Affiliations:** 1 Department of Respiratory Therapy, Fu Jen Catholic University Hospital, Fu Jen Catholic University, New Taipei City, Taiwan; 2 Institute of Emergency and Critical Care Medicine, National Yang Ming Chiao Tung University, No. 201, Sec. 2, Shih-Pai Road, Beitou District, Taipei, 112 Taiwan, R.O.C.; 3 School of Physical Therapy, Graduate Institute of Rehabilitation Sciences, Chang Gung University, Taoyuan, Taiwan; 4 Artificial Intelligence Development Center, Fu Jen Catholic University, New Taipei City, Taiwan; 5 Department of Nursing, Shin Kong Wu Ho-Su Memorial Hospital, Taipei, Taiwan; 6 Division of Pulmonary Medicine, Shin Kong Wu Ho-Su Memorial Hospital, Taiwan. No.95, Wenchang Rd., Shilin Dist., Taipei, 111 Taiwan, R.O.C.; 7 School of Medicine, College of Medicine, Fu Jen Catholic University, New Taipei City, Taiwan; 8 Division of Thoracic Surgery, Department of Surgery, Taipei Veterans General Hospital, Taipei, Taiwan

**Keywords:** Respiratory distress syndrome, Therapeutics

## Abstract

Many intensive care unit patients who undergo endotracheal extubation experience extubation failure and require reintubation. Because of the high mortality rate associated with reintubation, postextubation respiratory management is crucial, especially for high-risk populations. We conducted the present study to compare the effectiveness of oxygen therapy administered using high-flow nasal cannulae (HFNC) and noninvasive positive pressure ventilation (NIPPV) in preventing reintubation among patients receiving prolonged mechanical ventilation (PMV). This single-center, prospective, unblinded randomized controlled trial was at the respiratory care center (RCC). Participants were randomized to an HFNC group or an NIPPV group (20 patients in each) and received noninvasive respiratory support (NRS) administered using their assigned method. The primary outcome was reintubation within7 days after extubation. None of the patients in the NIPPV group required reintubation, whereas 5 (25%) of the patients in the HFNC group required reintubation (*P* = 0.047). The 90-day mortality rates of the NIPPV and HFNC groups (four patients [20%] vs. two patients [10%], respectively) did not differ significantly. No significant differences in length of RCC stay, length of hospital stay, time to liberation from NRS, and ventilator-free days at 28-day were identified. The time to event outcome analysis also revealed that the risk of reintubation in the HFNC group was higher than that in the NIPPV group (*P* = 0.018). Although HFNC is becoming increasingly common as a form of postextubation NRS, HFNC may not be as effective as NIPPV in preventing reintubation among patients who have been receiving PMV for at least 2 weeks. Additional studies evaluating HFNC as an alternative to NIPPV for patients receiving PMV are warranted.

ClinicalTrial.gov ID: NCT04564859; IRB number: 20160901R.

Trial registration: ClinicalTrial.gov (https://clinicaltrials.gov/ct2/show/NCT04564859).

## Introduction

Approximately 10–15% of intensive care unit (ICU) patients who undergo endotracheal extubation experience extubation failure and require reintubation^1^. This rate can even exceed 20% among patients with risk factors, including age more than 65 years, an underlying chronic cardiac or lung disease, and prolonged mechanical ventilation (PMV)^1,2^. PMV is associated with increased risks of reintubation^3^ and mortality^3–6^. Because of the high mortality rate associated with reintubation, postextubation respiratory management is crucial, especially for high-risk populations^7^. Noninvasive positive pressure ventilation (NIPPV) is widely used as a preventive measure against reintubation among high-risk populations, including patients with heart failure or obstructive lung diseases^8^. After extubation, NIPPV can be immediately applied as an early weaning strategy^9,10^, routinely applied for all patients at high risk of reintubation^11^, or applied for patients who develop respiratory distress^12^. A systematic review revealed that as a weaning strategy, NIPPV has advantages over invasive ventilation, including lower rates of weaning failure, reintubation, and mortality, and that the benefits of NIPPV for mortality were significantly greater in studies enrolling only patients with chronic obstructive pulmonary disease (COPD)^13^. Two meta-analyses revealed that early application of NIPPV can reduce reintubation rates; however, a subgroup analysis focusing on patients at high risk of reintubation has not yet been fully investigated^14,15^.

High-flow oxygen therapy can be administered using a nasal cannula^16^ or through a tracheostomy^17^. A high-flow nasal cannula (HFNC) can deliver up to 60 L/min of warm gas flow with adequate humidification (relative humidity of nearly 100%)^18,19^. Postextubation oxygen therapy with a HFNC has benefits for patients with acute hypoxemic respiratory failure^20^. A large-scale randomized controlled trial (RCT) reported that HFNC was noninferior to NIPPV for preventing reintubation and postextubation respiratory failure in patients at high risk of extubation failure^21^. However, to the best of our knowledge, the effectiveness of HFNC and NIPPV in preventing reintubation among patients receiving PMV have remained inconclusive. We conducted the present study to compare the effectiveness of HFNC and NIPPV in preventing reintubation among patients receiving PMV.

## Results

**Figure 1:**
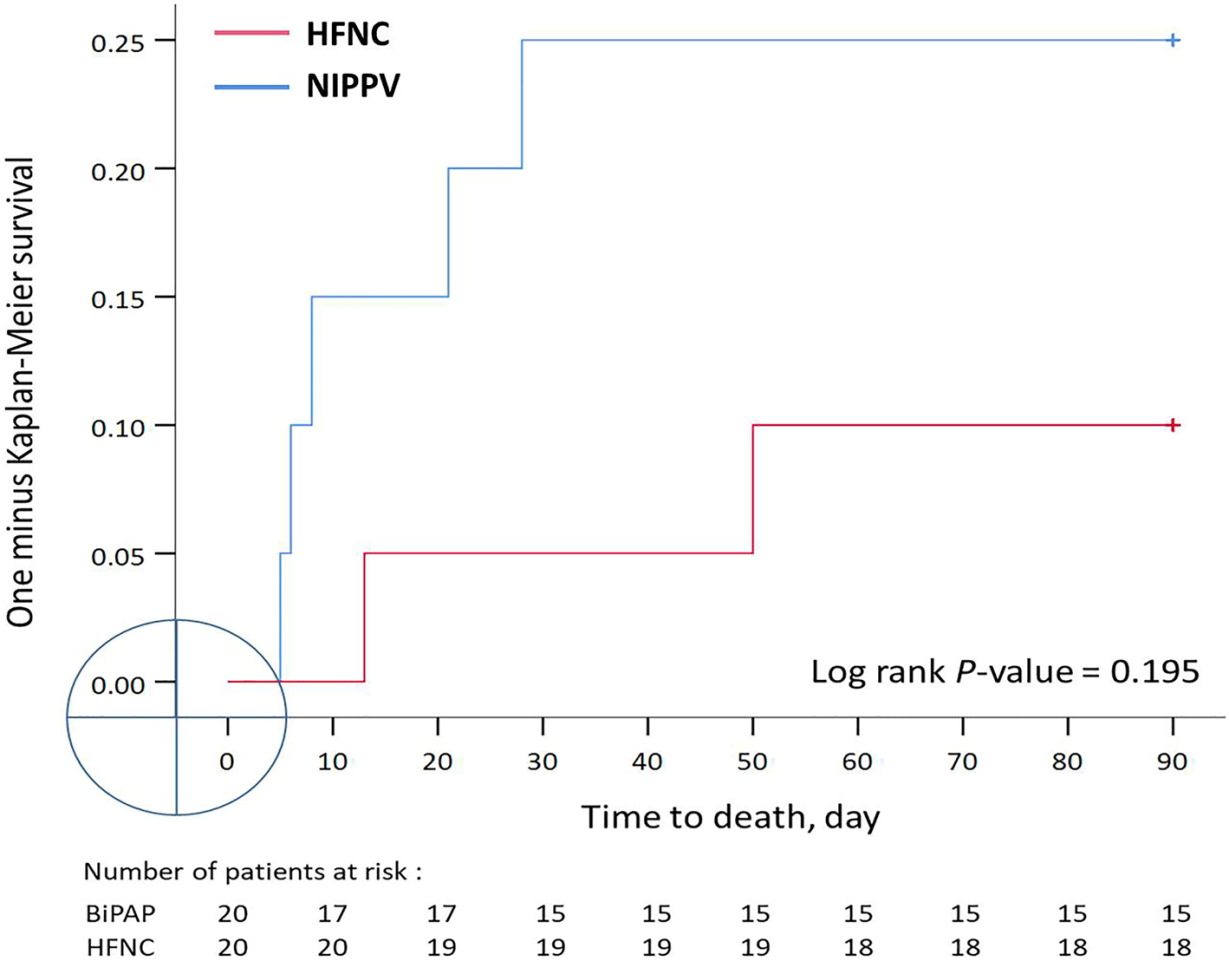
One minus Kaplan–Meier curves for mortality reintubation in the NIPPV and HFNC groups during the 90-day follow-up period. NIPPV: noninvasive positive-pressure ventilation; HFNC: high-flow nasal cannula.

A total of 40 patients were enrolled and assigned to the NIPPV and HFNC groups, with 20 patients in each group (see Supplementary Fig. S1 online). The mean ages of the NIPPV and HFNC groups were 74 ± 13 and 75 ± 11 years, respectively. Approximately half of the patients were male (65% and 50% in the NIPPV and HFNC groups, respectively). Most of the patients were recruited from the medical ICU (85% in both groups). The median numbers of high-risk factors for reintubation in the NIPPV and HFNC groups were 5.0 (interquartile range [IQR]: 4–6) and 4.5 (IQR: 4–6), respectively. The duration of mechanical ventilation in the NIPPV group was longer than that in the HFNC group (30 ± 9.8 vs. 26 ± 5.0 days, *P* = 0.079), a difference that reached borderline significance. Nevertheless, no significant between-group differences in any baseline characteristics were observed (Table 1). None of the patients in the NIPPV group required reintubation, whereas 5 (25%) of the patients in the HFNC group required reintubation within 72 h or 7 days (*P* = 0.047). The 90-day mortality rates of the NIPPV and HFNC groups (four patients [20%] vs. two patients [10%]) did not differ significantly. No significant differences in the length of RCC stay, length of hospital stay, time to liberation from NRS and ventilator-free days at 28-day were identified (Table 2). The analysis of time to event outcome also revealed that the risk of reintubation in the HFNC group was higher than that in the NIPPV group (*P* = 0.018; Fig. 1). The causes of reintubation were nasopharyngeal edema (*n* = 1), persistent postextubation respiratory failure (*n* = 3), and inability to clear secretions (*n* = 1).

**Table 1:** Characteristics of patients at inclusion.

Variables	NIPPV (*n* = 20)	HFNC (*n* = 20)	*P* value
Age, years	73 ± 13	74 ± 11	0.708
Male sex	13 (65)	10 (50)	0.523
APACHE II			
At ICU admission	17 ± 5.8	19 ± 8.3	0.405
At extubation	19 ± 4.7	18 ± 3.7	0.211
Route of admission			
Medical ICU	17 (85)	17 (85)	
Surgical ICU	3 (15)	3 (15)	
Cause of acute respiratory failure			
Pneumonia	7 (35)	12 (60)	0.205
Multiple trauma	1 (5.0)	2 (10)	1.000
Cardiac arrest	3 (15)	1 (5.0)	0.605
Shock	4 (20)	2 (10)	0.661
Cardiogenic pulmonary edema	1 (5.0)	1 (5.0)	1.000
Operation	2 (10)	1 (5.0)	1.000
Other	2 (10)	1 (5.0)	1.000
High risk factors for reintubation			
Age ≥ 65 years	16 (80)	16 (80)	1.000
Heart failure as primary indication for MV	12 (60)	6 (30)	0.111
COPD	3 (15)	5 (25)	0.695
APACHE II ≥ 12 at extubation	18 (90)	19 (95)	1.000
Body mass index ≥ 30 kg/m^2^	1 (5.0)	2 (10)	1.000
Airway patency problems	3 (15)	4 (20)	1.000
Inability to clear respiratory secretions	10 (50)	6 (30)	0.333
Difficult or prolonged weaning	18 (90)	15 (75)	0.407
Number of high-risk factors	5.0 [4.0–6.0]	4.5 [4.0–6.0]	0.242
Baseline (preextubation) physiological parameters			
Cuff leaks, mL	258 ± 119	301 ± 98	0.215
RSBI, breaths/m	83 ± 47	90 ± 41	0.628
RR, breaths/m	21 ± 5.0	21 ± 4.0	0.721
MAP, mmHg	87 ± 14	93 ± 11	0.125
Heart rate, beats/m	89 ± 19	90 ± 15	0.845
PaO_2_/FiO_2_	373 ± 150	316 ± 139	0.219
PaCO_2_, mmHg	38 ± 6.4	39.2 ± 8.3	0.620
pH	7.5 ± 0.05	7.5 ± 0.04	0.856
Length of ICU stay before inclusion, days	21 ± 7.3	18 ± 3.7	0.166
Duration of MV, days	30 ± 9.8	26 ± 5.0	0.079

Data are presented as frequencies (%), medians [interquartile range] or mean ± standard deviations.

NIPPV: noninvasive positive pressure ventilation; HFNC: high-flow nasal cannula; APACHE: acute physiology and chronic health evaluation; ICU: intensive care unit; COPD, chronic obstructive pulmonary disease; MV: mechanical ventilation; RSBI: rapid shallow breathing index; RR: respiratory rate; MAP: mean arterial pressure; PaO_2_: arterial oxygen tension; FiO_2_: fraction of inspiration O_2_.

**Table 2:** Reintubation and related outcomes.

Variables	NIPPV (*n* = 20)	HFNC (*n* = 20)	*P* value
Reintubation	0 (0)	5 (25)	0.047*
Mortality	4 (20)	2 (10)	0.331
Location of death			0.661
Respiratory care center	1 (5.0)	0 (0)	
Respiratory care ward (hospital)	3 (15)	2 (10)	
Length of respiratory care center stay, days	18 ± 10	17 ± 8.9	0.766
Length of hospital stay, days	56 ± 16	59 ± 23	0.666
Time to liberation from NRS, hours	15 ± 13	22 ± 17	0.181
Ventilator-free days at 28-day, days	26.8 ± 2.7	25.9 ± 6	0.548

Data are presented as frequencies (%) or means ± standard deviations. *P < 0.05.

NIPPV: noninvasive positive pressure ventilation; HFNC: high-flow nasal cannula; NIV: noninvasive ventilation, NRS: noninvasive respiratory support.

The results of the comparison of the patients’ physiological parameters and arterial blood gas analysis results at 1 h before and 2 h after extubation are presented in Table 3. None of the physiological parameters in the NIPPV group changed significantly from preextubation to postextubation. By contrast, the average respiratory rate and PaCO_2_ in the HFNC group increased from preextubation to postextubation (from 21 ± 3.7 to 24 ± 3.3 beat/min and from 39 ± 8.3 to 43 ± 9.9 mmHg, respectively). However, these changes in physiological parameters did not differ significantly between the groups (all *P* values for interaction > 0.05).

**Table 3:** Physiological parameters 1 hour before and 2 hours after extubation.

Variables	NIPPV (*n* = 20)		HFNC (*n* = 20)		Mean difference (95% CI)	*P* value for interaction
	Preextubation	Postextubation	Preextubation	Postextubation		
RR, breaths/m	21 ± 5.0	22 ± 4.6	21 ± 3.7	24 ± 3.3*	1.25 (− 1.80 to 4.30)	0.422
MAP, mmHg	87 ± 14	85 ± 12	93 ± 11	95 ± 9.3	4.0 (− 2.01 to 10.01)	0.192
HR, beats/m	89 ± 18	88 ± 16	90 ± 15	93 ± 14.3	4.6 (− 2.97 to 12.07)	0.236
PaO_2_/FiO_2_	373 ± 150	415 ± 143	316 ± 139	302 ± 127	− 56 (− 148 to 36)	0.232
PaCO_2_, mmHg	38 ± 6.4	39 ± 4.8	39 ± 8.3	43 ± 9.9*	3.31 (− 0.52 to 7.13)	0.091
pH	7.48 ± 0.05	7.47 ± 0.04	7.48 ± 0.04	7.47 ± 0.07	0.001 (− 0.03 to 0.03)	0.956

Data were presented as mean ± standard deviation.

*Indicated significant difference before and after extubation within one group.

NIPPV: noninvasive positive pressure ventilation; HFNC: high-flow nasal cannula; RR: respiratory rate; MAP: mean arterial pressure; HR: heart rate; PaO_2_: arterial oxygen tension; FiO_2_: fraction of inspiration O_2_.

## Discussion

**Figure 2:**
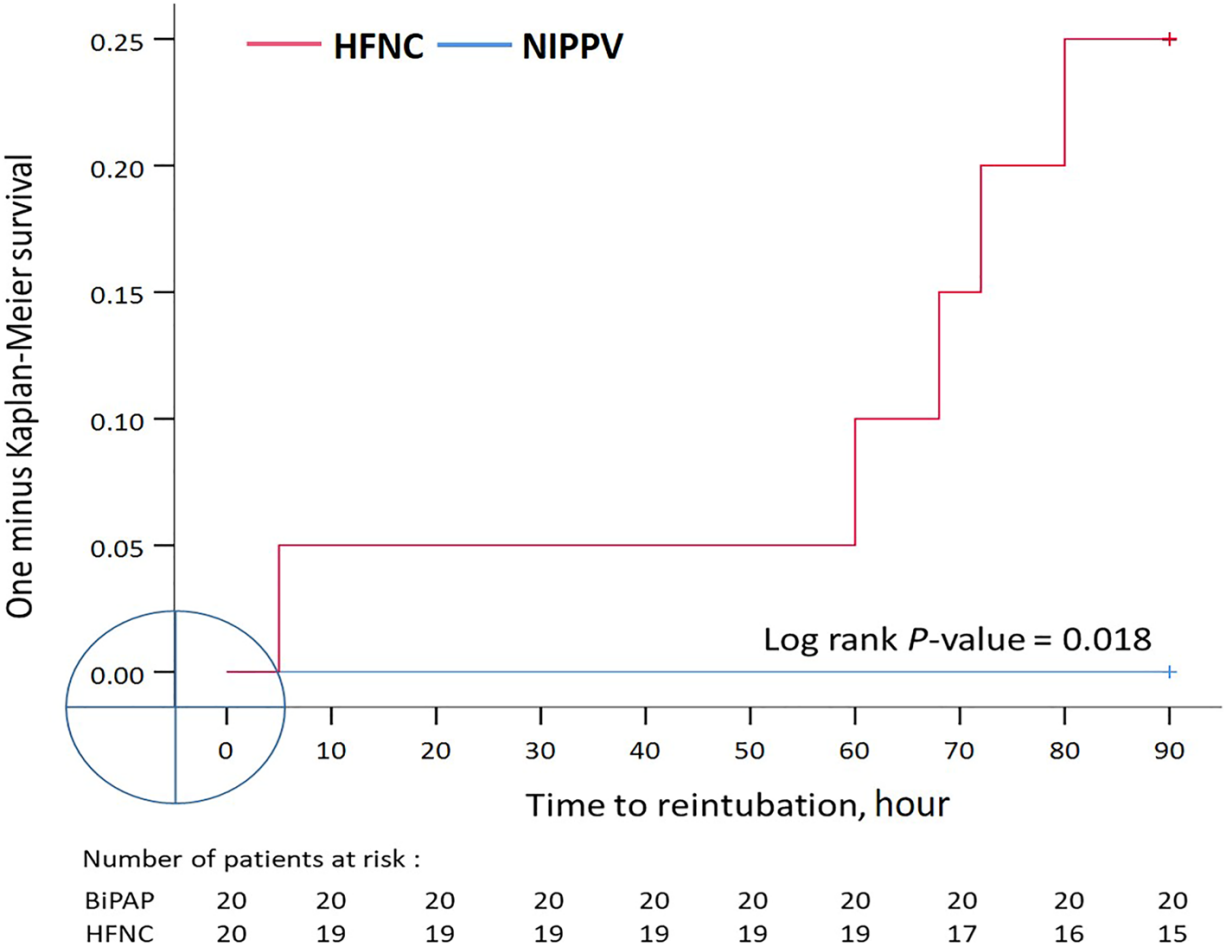
One minus Kaplan–Meier curves for reintubation in the NIPPV and HFNC groups during the 90-h follow-up period. NIPPV: noninvasive positive-pressure ventilation; HFNC: high-flow nasal cannula.

The results of this RCT indicate that patients with PMV who undergo HFNC have a higher intubation rate than do those who receive NIPPV. All the patients in this study had been receiving mechanical ventilation for more than 2 weeks (30 and 26 days on average in the NIPPV and HFNC groups, respectively). A total of 25% of the patients in the HFNC group required reintubation, whereas none of the patients in the NIPPV group were reintubated (Fig. 2). Three of the patients in HFNC group were reintubated because of persistent respiratory distress. One of the patients was reintubated because of laryngeal edema, and the final patient was reintubated because they were unable to clear airway secretions.

The mechanisms by which NIPPV has benefits to reduce reintubation rates include the following: (1) positive airway pressure can increase intrathoracic pressure, decreasing right ventricular preload and afterload; (2) adequate level of positive end-expiratory pressure (PEEP) increases functional residual capacity and prevents alveolar atelectasis; and (3) positive airway pressure can counterbalance hydrostatic forces leading to pulmonary edema and can help maintain airway patency^22–25^. Although HFNC results in lower rates of postextubation respiratory failure and reintubation than does conventional oxygen therapy (COT)^26,27^, delivering a sustained and consistent airway pressure to patients can be difficult. In the present study, one of the patients in the HFNC group was reintubated because of laryngeal edema. Although COT is the most common form of postextubation NRS, in recent years, NIPPV and HFNC are being increasingly used often for both management and prevention of postextubation respiratory failure, especially for patients receiving PMV^21,28^. NRS has been used prophylactically to minimize patients’ risk of requiring reintubation as well as their durations of MV and to improve the overall prognoses of patients at high risk of postextubation failure^29–32^. Thille et al. evaluated the effectiveness of NIPPV as rescue therapy for patients who experienced postextubation respiratory failure after receiving HFNC alone^28^. A combination of HFNC and NIPPV results in a lower reintubation rate than does HFNC alone, especially among patients with hypercapnia^28^. Patients aged ≥ 65 years who are receiving PMV are at a high risk of postextubation failure. Few studies have investigated the postextubation management of patients receiving PMV and HFNC, especially those who receive longer PMV day before they undergo extubation. Compared with the patients in the study by Hernandez et al., the patients in our study had a longer average duration of mechanical ventilation before extubation^21^. A previous report described that NIV can provide humidification through an active humidifier with a heated-wire respiratory circuit and is, therefore, superior to HFNC for preventing reintubation in patients at high risk of extubation failure^33^. Although we employed an active humidifier with a non–heated-wire respiratory circuit, the aforementioned result is consistent with the findings of our study. Casey et al. conducted a pragmatic, cluster–crossover trial involving 751 critically ill patients undergoing extubation from mechanical ventilation, and their results revealed that protocolized postextubation respiratory support with NIV or HFNC was not better able to prevent reintubation than usual care was^34^.

The Acute Physiology and Chronic Health Evaluation II scores of most patients in the present study were ≥ 12 on the day of extubation. Several risk factors for reintubation have been identified, and these factors may vary by the cause of reintubation^35^. In the present study, the reintubation rate of the patients receiving PMV who received HFNC was higher than that of the patients receiving PMV who received NIPPV also with a slight effect on mortality. The preextubation-to-postextubation changes in the patients’ physiological parameters and arterial blood gas analysis results were comparable between the groups. However, HFNC was associated with a higher respiratory rate and PaCO_2_ level after extubation. Tan et al. reported that although HFNC in patients with severe hypercapnia respiratory failure did not increase the treatment failure rate compared with NIV, HFNC increased their tolerance for the treatment and the comfort of the treatment. Moreover, Tan et al. reported that the PaCO_2_ level in the HFNC group was higher than that at 1 h after extubation, which is consistent with our results (Table 3). However, Tan et al. also discovered that the PaCO_2_ level at 24 h and 48 h after extubation did not significantly differ in the HFNC group^36^.

The groups’ average length of hospital and RCC stay and time to liberation from NRS were comparable. The in-RCC mortality, in-hospital mortality rates and numbers of ventilator-free days were similar between the groups. Although the morbidity and mortality risks related to reintubation could not be determined in the present study, reintubatation with or without side effects is a preferable outcome to mortality.

This study has several limitations. First, the patients and attending teams could not be blinded because of the nature of the treatments. The researchers were excluded from clinical decisions to minimize bias; however, completely eliminating bias was impossible. Second, the NIPPV provided a higher level of PEEP than did the HFNC. Because we did not measure the actual FiO_2_ delivered to the patients, we could not determine whether the comparable PaO_2_/FiO_2_ ratios of the groups were attributable to the HFNCs providing a higher FiO_2_. Third, an HFNC is an open-loop system, and measuring delivered airway pressure and tidal volume is often difficult due to the device’s limitations. Therefore, the average CO_2_ clearance of the HFNCs was not obtained. Fourth, this was a single-center study with a small sample size, which may have limited the quality of our statistical analyses. Fifth, we did not conduct long-term follow-up. However, Nagata et al. recently reported that using HFNC for patients with stable hypercapnic COPD reduced the incidence of acute exacerbations.

Larger prospective RCTs on this topic should be conducted in the future. Although HFNC is increasingly being used often as a form of postextubation NRS, HFNC may not be as effective as NIPPV in preventing reintubation among patients who have been receiving PMV for at least 2 weeks. NIPPV may be used as rescue therapy for patients receiving PMV who experience postextubation respiratory failure after receiving HFNC alone. Additional studies evaluating HFNC as an alternative to NIPPV for patients receiving PMV are warranted.

## Methods

### Study design

This single-center, prospective, nonblinded RCT and equivalent RCT that compared NIPPV (intervention group) and HFNC (control group) after extubation was conducted between January 2017 and December 2020 at the respiratory care center (RCC) of Shin Kong Wu Ho-Su Memorial Hospital in Taipei, Taiwan. The study was approved by the Institutional Review Board of Shin Kong Wu Ho-Su Memorial Hospital (IRB number: 20160901R), and written informed consent was obtained from all the participants or their relatives. The trial was registered at ClinicalTrials.gov (NCT04564859 25/09/2020).

### Participants

Patients aged more than 20 years who were ready for extubation, had received mechanical ventilation for more than 6 h per day for at least 14 consecutive days, and who had been transferred from the ICU to the RCC were immediately enrolled into this study. The exclusion criteria for this study were tracheostomy, do-not-intubate status, pregnancy, neuromuscular diseases, and unplanned extubation.

In Taiwan, an integrated delivery system was launched to reduce the average length of ICU stay and improve the quality of care of patients with PMV^37^. Patients receiving mechanical ventilation are transferred to subacute care facilities, such as RCC or respiratory care ward, on the basis of the number days for which they have been receiving mechanical ventilation and their clinical status^37^.

### Intervention

The clinical weaning protocol involved a daily evaluation of weaning readiness up to the time of extubation, and the readiness was determined on the basis of the following criteria: recovery from the precipitating illness; respiratory measures of PaO_2_/FiO_2_ > 150 with FiO_2_ ≤ 0.4, PEEP < 8 cmH_2_O, and pH > 7.35; the absence of electrocardiographic signs of myocardial ischemia; no requirement for vasoactive drugs or a requirement for only low-dose dopamine (< 5 µg/kg/min; heart rate < 140 b/min, hemoglobin > 8 g/dL, temperature < 38 °C); no need for sedatives; the presence of a respiratory stimulus; and appropriate spontaneous cough. Patients who met these criteria underwent a spontaneous breathing trial with either T-piece or pressure support of 6–8 cmH_2_O for 30 to 120 min according to the patient’s condition. The following factors indicated respiratory failure: a respiratory rate > 35 b/min, SpO2 < 92%, exhaled tidal volume < 4 mL/kg, heart rate > 140 b/min or 25% above baseline or < 60 b/min, blood pressure increased to 40 mm Hg above the baseline, worsening agitation, and anxiety or discomfort despite reassurance.

After undergoing extubation, each patient was randomly assigned to the HFNC and NIPPV groups (in a 1:1 ratio) and received noninvasive respiratory support (NRS) administered using their assigned method. Randomization was achieved through the use of a website (http://randomization.com). Each group assignment was provided in a consecutively numbered, sealed, opaque envelope. The patients in the HFNC group received continuous flow of oxygen through a nasal cannula (Optiflow, Fisher & Paykel Healthcare, Auckland, New Zealand) with a high-flow oxygen system (Airvo^2^, Fisher & Paykel Healthcare, Auckland, New Zealand). The initial flow rate was set to 50 L/min with subsequent adjustments to maintain adequate gas exchange. To provide adequate airway humidity, the gas temperature was set to 34 °C or 37 °C according to each patient’s airway secretion condition. NIPPV was delivered using a Trilogy 202 ventilator (Philips Respironics, Murraysville, PA, USA) with a facemask (Mirage Quattro, ResMed, Sydney, Australia). The inspiratory pressure and end-expiratory pressure were set to 12–16 and 4–6 cmH_2_O, respectively, to maintain a tidal volume of 6–10 mL/kg.

### NRS weaning protocol

All participants were screened for weaning readiness daily according to the following criteria: (1) pH value of ≥ 7.35; (2) oxygen saturation (SpO_2_) of > 90% at a fraction of inspired oxygen (FiO_2_) of ≤ 0.5; (3) respiratory rate of ≤ 25 breaths/min; (4) heart rate of ≤ 120 beats/min; (5) systolic blood pressure of ≥ 90 mmHg; and (6) no signs of respiratory distress, such as agitation, diaphoresis, paradoxical respiration, accessory muscle recruitment, or anxiety.

### Failure criteria

Postextubation respiratory failure was defined as follows: (1) lack of improvement in pH or in the partial pressure of carbon dioxide (PaCO_2_) under NRS; (2) decrease in SpO_2_ to ≤ 85% despite FiO_2_ of ≥ 0.5; (3) persistent or worsening signs of respiratory muscle fatigue; (4) copious secretions that could not be adequately cleared; (5) changes in mental status; (6) acute upper airway obstruction; and (7) hemodynamic instability. Patients who fulfilled these criteria were considered to be reintubated. The final decision to reintubate was made by the treating physician.

### Outcome measurement

The primary outcome was reintubation within 72 h after extubation. The secondary outcomes were reintubation within 7 days of extubation, changes in physiological parameters and arterial blood gas analysis results, time to liberation from NRS, duration of respiratory support, length of RCC and hospital stay, ventilator-free days at 28-day and 90-day mortality. Successful liberation from NRS was defined as the time point at which a patient was alive and free of NRS (HFNC or NIPPV) for more than 48 h.

### Statistical analysis

The primary outcome of this study was the reintubation rate. We assumed the reintubation rates of the HFNC and NIPPV groups to be 50% and 10%, respectively. A sample size of 20 for each group was required to achieve an alpha level of 5% (two tailed) and power of 80%. Sample size calculation was conducted using G*Power, version 3.1.9.4 (University of Kiel, Kiel, Germany). The clinical characteristics and outcomes of the patients in the NIPPV and HFNC groups were compared using Fisher’s exact test (for categorical variables) or an independent-samples *t* test (for continuous variables). The number of high-risk factors for reintubation in each group is expressed as a median with an interquartile range, and the numbers were compared using a Mann–Whitney *U* test. The cumulative incidence of reintubation and mortality within 90 days was estimated using the Kaplan–Meier method, and a log-rank test was used to compare the groups. Finally, the preextubation-to-postextubation changes in the physiological parameters of the NIPPV and HFNC groups were compared using a generalized estimating equation (GEE). The link function was identity, the distribution was normal, and the working correlation matrix was exchangeable. The robust standard error was used in the GEE model to test the intercept, main effects of time (preextubation vs. postextubation) and group, and the interaction effect between time and group. In addition, preextubation-to-postextubation changes within each group were tested using the contrast within the GEE model. All the tests were two-tailed, and a *P* value of < 0.05 was considered statistically significant. Statistical analyses were conducted using IBM SPSS (version 25.0 for Windows, Chicago, IL, USA).

### Ethics approval and consent to participate

We conducted the trial in accordance with good clinical practice guidelines and the Declaration of Helsinki. The study was approved by local institutional review committees (IRB number: 20160901R). Informed written consent was obtained from all participants.

## Supplementary Information

Supplementary Figure S1.

Supplementary Legends.

## Data Availability

The datasets used and/or analysed during the current study available from the corresponding author on reasonable request.
