# Supplementary figures and images for: Effectiveness of high-flow nasal cannulae compared with noninvasive positive-pressure ventilation in preventing reintubation in patients receiving prolonged mechanical ventilation
Source: Sci Rep. 2023 Mar 22;13:4689. doi: 10.1038/s41598-023-31444-8 (PMC10033681; doi:10.1038/s41598-023-31444-8)

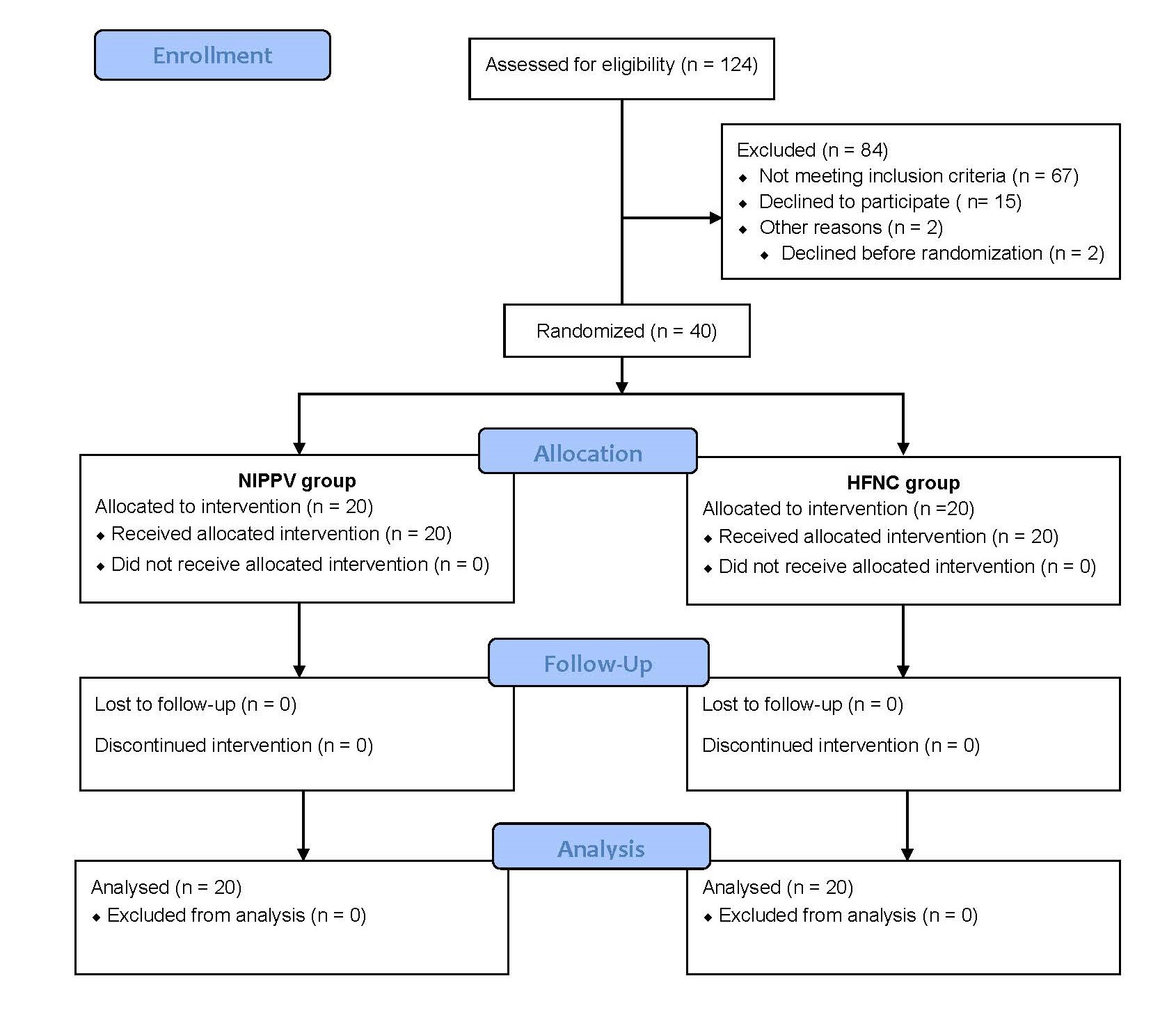

Supplement: Supplementary file 1 — Supplementary Figure S1. [file 41598_2023_31444_MOESM1_ESM.jpg]
