# Supplementary material for: Effectiveness of high-flow nasal cannulae compared with noninvasive positive-pressure ventilation in preventing reintubation in patients receiving prolonged mechanical ventilation
Source: Sci Rep. 2023 Mar 22;13:4689. doi: 10.1038/s41598-023-31444-8 (PMC10033681; doi:10.1038/s41598-023-31444-8)
Supplement: Supplementary file 2 — Supplementary Legends. [file 41598_2023_31444_MOESM2_ESM.docx]

**Supplementary legends**

**Fig. S1**. CONSOR flow diagram. NIPPV: noninvasive positive pressure ventilation; HFNC: high flow nasal cannula.
